# The microbiome in pancreatic diseases: Recent advances and future perspectives

**DOI:** 10.1177/2050640620944720

**Published:** 2020-07-23

**Authors:** Christoph Ammer-Herrmenau, Nina Pfisterer, Mark FJ Weingarten, Albrecht Neesse

**Affiliations:** Department of Gastroenterology, Gastrointestinal Oncology and Endocrinology, University Medical Centre Goettingen, Goettingen, Germany

**Keywords:** Pancreatic cancer, pancreatitis, microbiome, 16S rRNA sequencing, metagenomic sequencing

## Abstract

The human microbiota exerts multiple physiological functions such as the regulation of metabolic and inflammatory processes. High-throughput sequencing techniques such as next-generation sequencing have become widely available in preclinical and clinical settings and have exponentially increased our knowledge about the microbiome and its interaction with host cells and organisms. There is now emerging evidence that microorganisms also contribute to inflammatory and neoplastic diseases of the pancreas. This review summarizes current clinical and translational microbiome studies in acute and chronic pancreatitis as well as pancreatic cancer and provides evidence that the microbiome has a high potential for biomarker discovery. Furthermore, the intestinal and pancreas-specific microbiome may also become an integrative part of diagnostic and therapeutic approaches of pancreatic diseases in the near future.

## Introduction

The human body is colonized by individual sets of microbes. In fact, prokaryotic cells outnumber eukaryotic cells within the human body by far. The gastrointestinal tract alone harbours more than 10^14^ microorganisms that carry over five million genes maintaining numerous physiological functions such as metabolic pathways, modulation of the immune system, vitamin production, regulation of digestion and modulation of intestinal architecture.^1^ Interestingly, the bacterial composition differs substantially between healthy individuals depending on age, sex, genetic features, dietary habits, physical exercise and multiple other factors.^2^ However, despite the observed interindividual differences, the metabolic pathways of gut microbes are highly stable within the healthy population.^3^

Alterations in the bacterial composition, also known as dysbiosis, contribute to various gastrointestinal and metabolic disorders such as inflammatory bowel diseases, colorectal cancer, obesity and diabetes.^2^ Over the past few years, increasing evidence has emerged showing the orointestinal microbiome impinges on other organs such as the heart, liver and pancreas.

Inflammatory and neoplastic diseases of the pancreas are highly challenging for clinicians with many open questions regarding diagnosis and clinical management. For instance, early estimation of the severity of pancreatitis as well as the use of antibiotics for pancreatitis patients remains a controversial topic. Recent data from several microbiome studies have started to elucidate the role of the microbiome in pancreatic diseases. For instance, it was recently shown that pancreatic tumours harbour their own characteristic microbiome that is entirely different to normal pancreas tissue, modulates the tumoral immune system^4^ and potentially impedes chemotherapy response.^5^ Despite an increasing body of evidence that the microbiome co-evolves and significantly changes during the course of disease, most studies have not yet conclusively answered the question of whether microbes alone are sufficient to cause diseases such as acute or chronic pancreatitis (CP). Here, we aim to summarize clinically and translationally relevant data regarding the impact of the microbiome on pancreatic diseases. Furthermore, we highlight methodological and experimental strengths and pitfalls of modern microbiome research.

## Acute pancreatitis

The incidence of acute pancreatitis (AP) ranges from 13–45/100,000 and patients often require hospital admission. Regardless of the aetiology, systemic inflammatory response syndrome (SIRS) in AP patients can cause hypovolemia and impaired microcirculation with subsequent end-organ damage such as renal, lung and circulatory failure with subsequent gut mucosal ischemia. Particularly, the latter results from vasoconstriction of the splanchnic vessels and can lead to a disturbed integrity of the gut barrier and translocation of intestinal bacteria. A meta-analysis by Wu et al. analysed 18 studies that focused on dysfunction of the gut barrier in patients with AP. The authors found that almost 60% of patients suffer from a condition called leaky gut.^6^ The circulating bacteria have the potential to aggravate consecutive SIRS symptoms. Although current guidelines do not recommend routine use of prophylactic antibiotics in patients without accompanying cholangitis or infected necrosis,^7^ a subpopulation of patients might benefit from early antibiotic treatment.^8^,^9^ To further characterize relevant prognostic subpopulations of AP patients, Zhu and colleagues performed 16S marker gene sequencing of stool samples and discovered an increased dysbiosis in the course of deteriorating AP that correlated with systemic inflammation and gut barrier dysfunction.^10^

**Figure 1.**
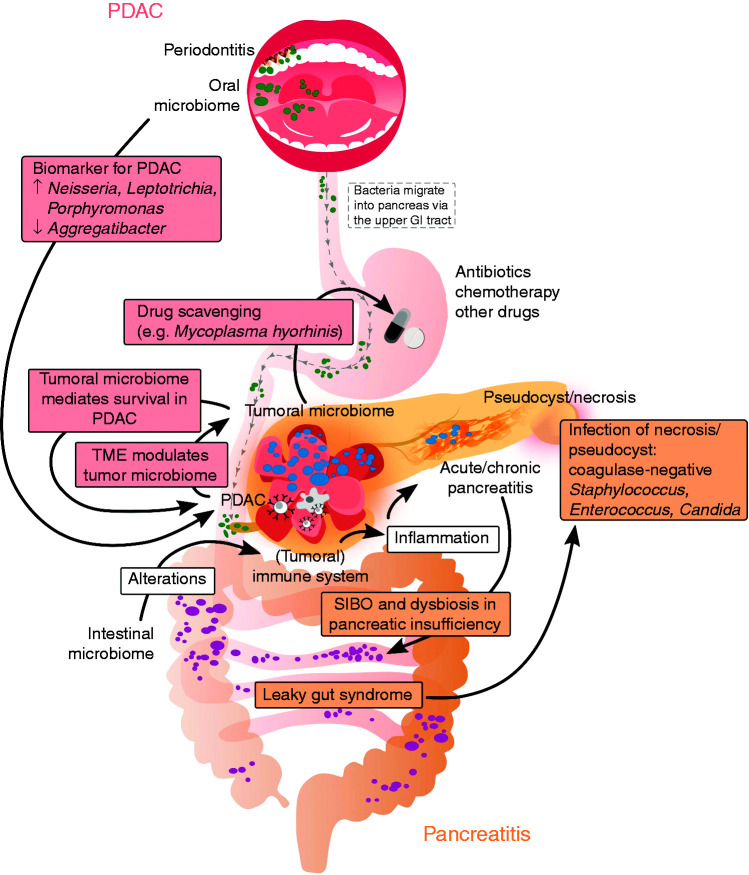
Key findings and current perceptions of the orointestinal microbiome in pancreatic ductal adenocarcinoma (PDAC) and acute and chronic pancreatitis.

One feature of moderately severe and severe AP is the development of infected necrosis. Circulating microbes likely cause infection of necrotic collections thus leading to a higher morbidity and mortality. A meta-analysis of 71 studies including nearly 7000 patients concluded that the mortality of patients with infected necrosis is more than twice as high as the mortality of those with sterile necrosis or pseudocysts (28% vs. 13%).^11^ According to current knowledge, bacteria translocate to necrotic collections from the small bowel rather than from the colon (Figure 1).^12^ These findings are in line with additional translational experiments showing the migration of fungi and bacteria into the pancreas via the upper gastrointestinal tract.^13^,^4^ Culture-based analyses revealed that *Enterococcus* spp., coagulase-negative *Staphylococcus* and *Candida* spp. are commonly found in walled-off necrosis (WON) samples obtained by endoscopic puncture and aspiration.^14^,^15^ A potential limitation of this retrospective analysis is that less than half of the samples showed a positive culture result, and most patients were treated with antibiotics before draining the WON. All three genera are also known as commensals rather than being the main infecting germs, simply selected by antibiotic treatments. Comprehensive high throughput approaches such as marker gene or metagenomic sequencing are still missing. These appealing techniques could expand our knowledge about the suspected polymicrobial commensals of necrosis and answer remaining questions such as which necrotic formation requires an early drainage and which will resolve spontaneously.

## Chronic pancreatitis

CP is a fibro-inflammatory disease with a considerable morbidity and mortality. It is characterized by a variety of local and systemic complications such as chronic pain, pancreatic exocrine insufficiency (PEI), diabetes and pseudocyst formation. Dysbiosis is a known and expected phenomenon in progressive CP. The pancreas physiologically regulates the gut microbiome by synthesizing anti-microbial peptides, bicarbonate and digestive enzymes. If the secretion of pancreatic juice is impaired due to PEI, small intestinal bacterial overgrowth (SIBO) and gut dysbiosis are logical consequences (Figure 1). This hypothesis is supported by a recent landmark study in which stool samples of more than 1700 individuals without a history of pancreatic disease were analysed for elastase and 16S rRNA sequencing. Frost et al. could show that reduced elastase is the most relevant host factor associated with alterations in the intestinal microbiome.^16^ And indeed, a recent meta-analysis by Capurso et al. with 336 patients revealed that one-third of patients with CP have SIBO.^17^ For the clinical practice, it is therefore important to consider SIBO in patients with CP. The symptoms of bacterial overgrowth are very similar to those of PEI, such as diarrhoea, flatulence and bloating. Therefore, a fasting glucose hydrogen breath test should be performed in patients with clinically relevant PEI whose symptoms are unresolved by adequate doses of pancreatic enzyme replacement therapy.^18^

Apart from small intestinal dysbiosis, changes in the colon microbiota have recently been described. Using β-diversity, the difference in microbial communities between individuals, the microbiome of CP patients with diabetes could be distinguished from those without diabetes and healthy controls (HC).^19^ In contrast, α-diversity describes the number of different species within an individual microbial system and was shown to be lowest in patients with CP and diabetes compared to CP patients without diabetes and HC.^19^ In particular, *Faecalibaterium prausnitzii* was greatly reduced. This bacterium has important functions in maintaining intestinal barrier homeostasis and shifting the mucosal immune system towards a more tolerant and anti-inflammatory mode.^20^ However, as pointed out earlier, it remains unclear whether dysbiosis is a result of metabolic or inflammatory conditions or rather causally triggers the disease course and potential complications.

## Pancreatic ductal adenocarcinoma

Pancreatic ductal adenocarcinoma (PDAC) is the most common malignancy of the pancreas with an extremely poor prognosis and a 5-year survival rate of around 8%.^21^ By 2030, calculations predict that PDAC will be the second leading cause of cancer-related deaths after lung cancer.^22^ Reasons for this poor prognosis are a lack of early clinical symptoms, high recurrence rate after resection, resistance to common chemotherapy and early metastatic spread.^23^ Therefore, there is an urgent need to discover new biomarkers that may aid early diagnosis and more efficiently treat this dismal disease.

For instance, it is known that periodontitis is a risk factor for the development of PDAC (Figure 1).^24^ A series of case control studies have indicated that the oral microbiome is distinct from HC and CP patients (Table 1). Although the authors describe a decent sensitivity and specificity for the observed microbiome alterations, indicating potential value as diagnostic tool, these studies show substantial heterogeneity and controversial results when compared to each other. To this end, the oral microbiome was analysed using various specimen such as salivary,^25–29^ oral wash samples,^30^ blood antibodies against oral bacteria^24^ or tongue swabs.^31^ Some authors found the genera *Neisseria*, *Leptospira* and *Porphyromonas* to be increased and *Aggregatibacter* decreased in specimens of PDAC patients (Figure 1). In contrast, other studies claim the opposite (Table 1).

**Table 1.** Summary of current studies regarding the oral microbiome as non-invasive biomarker for pancreatic cancer.

Year of publication	Authors	Study design	Method	Number of patients	Change in bacterial composition	References
2012	Farrell et al.	Case control	Real-time quantitative PCR of saliva	38 PDAC, 38 HC, 27 CP	*Neisseria elongata*, *Streptococcus mitis*	23

2013	Michaud et al.	Prospective population-based	Blood samples, immunoblot array	405 PDAC, 416 HC	*Porphyromonas gingivalis* ATTC 53978	22

2013	Lin et al.	Pilot study	16S RNA sequencing of oral wash samples	13 PDAC, three CP, 12 HC	*Bacteroides* increased, *Corynebacterium* and *Aggregatibacter* decreased in PDAC	28

2015	Torres et al.	Cross-sectional	16S RNA sequencing and quantitative PCR of saliva	Eight PDAC, 22 HC	*Leptotrichia* and *Porphyromonas* increased, *Neisseria* and *Aggregatibacter* decreased in PDAC	26

2016	Fan et al.	Case control	16S RNA sequencing of saliva	361 PDAC, 371 HC	*Porphyromonas gingivalis* and *Aggregatibacter actinomycetemcomitans* increased, *Leptotrichia* decreased in PDAC	24

2017	Olson et al.	Case control	16S RNA sequencing of saliva	40 PDAC, 39 IPMN, 58 HC	*Streptococcus* increased, *Haemophilus* and *Neisseria* decreased in PDAC	25

2019	Lu et al.	Case control	16S RNA sequencing of tongue swabs	30 PDAC, 25 HC	*Leptotrichia*, *Fusobacterium*, *Rothia*, *Actinomyces*, *Corynebacterium*, *Atopobium*, *Peptostreptococcus*, *Catonella*, *Filifactor*, *Campylobacter*, *Moraxella* and *Tannerella* increased, *Haemophilus*, *Porphyromonas* and *Paraprevotella* decreased in PDAC	29

2020	Vogtmann et al.	Case control	16S RNA sequencing of saliva	273 PDAC, 285 HC	*Enterobacteriaceae*, *Lachnospiraceae* G7, *Bacteroidaceae* or *Staphylococcaceae* increased and *Haemophilus* decreased in PDAC	27

CP: chronic pancreatitis; HC: healthy controls; PCR: polymerase chain reaction; PDAC: pancreatic ductal adenocarcinoma; IPMN: intraductal papillary mucinous neoplasm.

Interestingly, oral microbes may be directly implicated in the initiation and progression of PDAC. Pushalkar and colleagues were able to demonstrate that bacteria can migrate from the gut into the tumour.^4^ Using 16S rRNA sequencing the authors provide convincing evidence that the tumour harbours its own microbiome, which is completely distinct from the microbial composition of normal pancreas tissue. Particularly, the genera *Pseudomonas* and *Elizabethkingia* are highly abundant in tumours. Furthermore, these intratumoral bacteria are capable of modulating the tumour immune system by activating Toll-like receptors, supporting a more tolerant microenvironment. Hence, the tumoral microbiome seems to favour tumour progression (Figure 1). This hypothesis was also supported by data of Requilme et al. who compared tumoral and intestinal microbiota from short-term PDAC survivors (STS) with those of long-term survivors (LTS). Half of the STS lived around 1.5 years whereas half of the LTS died after 5–10 years. It is remarkable that both groups matched concerning age, gender, stage, past therapies and their genetic aberrations but differed significantly regarding their tumoral microbiome. Three genera, *Pseudoxanthomonas*, *Saccharopolyspora* and *Streptomyces*, and a species called *Bacillus clausii* were identified and could predict the prognosis of PDAC patients with high reliability.^23^ Furthermore, the authors showed that faecal microbiome transfer from LTS reduced tumour growth in mice compared to STS. Mechanistically, the authors postulate that the transplanted stool microbiome alters the tumoral immune system. LTS harbour a higher α-diversity and more cytotoxic T-cells in the tumour tissue compared to STS. In addition, a recent publication showed that bacteria are predominantly located intracellularly in tumour and immune cells and might thus directly affect tumour biology.^32^

Using marker gene sequencing for fungal DNA, a recent publication indicated that the mycobiome, especially *Malassezia* spp., exerts a tumour-promoting effect as well.^13^ However, marker gene sequencing has its limitations as it can only provide information of one special domain (bacteria or fungi) and exclude other highly interesting microbes such as viruses and bacteriophages. Furthermore, this technique can only identify microbes whose genomes are already included in a reference database. De novo assembly and detection of unknown microbes is only feasible with metagenomic analysis.^33^ Apart from that, highly host-contaminated samples such as tumoral tissue are problematic for this method and therefore require deep sequencing to obtain sufficient DNA sequences for microbiome analysis. The advantages and drawbacks of 16S rRNA compared to metagenomic sequencing are summarized in Table 2.

**Table 2.** Comparison of 16S rRNA and metagenomic sequencing.

	Advantages	Disadvantages
Marker gene sequencing (e.g. 16S rRNA sequencing for bacteria)	Fast, less complicated, cheaper library preparation and analysis	PCR introduces amplification bias, thus interferes with abundance analysis
	Suitable for highly host-contaminated samples and samples with low biomass	Choice of primers and variable regions have a huge influence of taxonomic
	Well-established bioinformatic tools	No possibility of de novo assembly
		Limited information at species level, best resolution at genus level

Metagenomic (whole genome sequencing)	Reliable abundances analysis	More expensive and labour-intensive library preparation and analysis
	Resolution to species and strain level	Deep sequencing is required due to host DNA contamination
	Possibility of de novo assembly	For example, viruses are not well annotated by widely used analysis workflows
	No PCR bias	
	Provides information of all sequenced and characterized microbes (bacteria, fungi, viruses, archaea)	
	Better resolution for functional profiling	

PCR: polymerase chain reaction; rRNA: ribosomal ribonucleic acid.

One hallmark feature of PDAC is the high resistance against most chemotherapies. Geller et al. reported that intratumoral bacteria are able to inactivate gemcitabine by expressing a long isoform of the enzyme cytidine deaminase (CDD_L_).^5^ Notably, this enzyme is mainly expressed by *Gammaproteobacteria* (e.g. *Mycoplasma hyorhinis*) and thus rapidly inactivates gemcitabine. Notably, this effect could be abrogated by co-treatment with the antibiotic ciprofloxacin. These findings are in line with the results of Pushalkar et al. and Relquime et al. who identified genera such as *Pseudomonas,* which belongs to the class of *Gammaproteobacteria*.^4^,^23^ Moreover, emerging evidence suggests the microbiome seems to play a crucial role in the response to targeted therapies in cancer patients. A series of high-ranking publications has recently indicated that checkpoint inhibitors require a distinct gut microbiome to fully execute their anticancer effects. Dysbiosis, for example mediated by antibiotic treatment, impairs the anti-tumoral efficacy of these anticancer drugs.^34–36^ Particularly, *Akkermansia muciniphila*, *Bacteroides fragilis*, *Bifidobacterium* spp. and *Faecalibacterium* spp. enhance antitumoral efficacy.

## Pancreatic surgery

The orointestinal microbiota not only has an influence on the common pathologies of the pancreas but also shows an association with postoperative complications. A recent prospective pilot study, in which the authors analysed stool samples before and after pancreas surgery, revealed that distinct microbiome patterns correlated with medical and surgical complications such as pulmonary embolism, infections as well as pancreatic fistulas and delayed gastric emptying.^37^ Interestingly, the microbiome was able to predict the likelihood of complications before surgery in some patients, whereas other individuals underwent alterations of their intestinal microbiome after surgery that resulted in a higher rate of complications. The main limitation of this study is the small number of included patients. Therefore, further investigations must be conducted to confirm these preliminary results.

## Methodological pitfalls of modern microbiome techniques

The majority of studies employed 16S rRNA marker gene sequencing. The gene encoding for 16S rRNA is about 1500 bps long and forms part of the small 30S subunit of prokaryotic 70S ribosomes. It consists of nine variable regions next to the highly conserved primer binding sites. Using next-generation sequencing most reads have around 150–300 bps, thus making it impossible to sequence the entire 16S rRNA gene in one run. Depending on the variable region for the selected pre-processing polymerase chain reaction (PCR) primers, results can widely vary and lead to different taxonomic classifications as demonstrated by the contradicting results of the presented studies (Table 1). A preferable but more expensive alternative is the metagenomic approach. Here, total DNA is sequenced and a subsequent PCR is not required for library preparation. Furthermore, metagenomic analysis has a better resolution at the level of species or even strains (Table 2).^33^

## Conclusion

The sequencing of 16S marker gene and metagenomics has revolutionized clinical and translational microbiome research. Recent efforts have attempted to exploit these techniques for diagnostic, predictive and prognostic biomarker discovery in inflammatory and neoplastic pancreatic disorders. Interesting associations between certain microbiome patterns and disease outcomes (e.g. severity of pancreatitis, survival of PDAC patients) have been identified recently. However, in most instances it remains unclear whether the microbiome causally promotes or attenuates pancreatic diseases or rather co-evolves with a particular disease. Importantly, the advent of high-throughput sequencing techniques from low abundance samples not only allows the investigation of the intestinal microbiome, but also gives compelling insights into organ- and cell-specific bacteria (e.g. intracellular accumulation of bacteria in tumour and immune cells). Rigorous experimental conditions including extensive HC samples and controls as well as selection of the appropriate sequencing method will yield fascinating results in the near future. Clinical trials such as the PANDEMIC study (NCT04274972) and LyRICX (NCT03764553) will further illuminate the role of the microbiome in PDAC and other cancer entities regarding surgical outcome and response to chemotherapy. In summary, the orointestinal microbiome has the potential to become a non-invasive diagnostic and prognostic biomarker for PDAC; however, further in-depth investigations using the more reliable method of metagenomic sequencing in well-characterized patient populations are required.
